# The Medical Reading Society, Bristol

**Published:** 1907-09

**Authors:** L. M. Griffiths


### THE MEDICAL READING SOCIETY, BRISTOL.

BY

L. M. Griffiths, M.R.C.S. Eng., L.R.C.P. Ed.

---

Rarely will it be possible to chronicle the doings for a hundred years of a society that has never at any time had a larger membership than twelve. But as the opportunity has recently occurred in this city, such an event should not be passed over without some comment, as the history of a Medical Society during such a long period cannot fail to bring out many interesting points.

On March 28th, 1807, some medical men of Bristol decided to form "The Medical Reading Society, for the purpose of promoting medical knowledge and a friendly intercourse among its members, and for purchasing medical books." Some practical rules, very similar to those of most book or magazine clubs, received the approval of these eleven members :—[1]

THOMAS JERMYN, Surgeon and Apothecary, 17 Queen Square.

HENRY DANIEL, Surgeon, 52 Queen Square.

RICHARD EDGELL, Surgeon, 68 College Street.

BENJAMIN SPENCER, Surgeon and Apothecary, Paul Street, Kingsdown.

WILLIAM MORTIMER.

ROBERT LAX.

BENJAMIN GUSTAVUS BURROUGHS, Apothecary, Portland Place, Clifton.

JOSEPH MAURICE, Apothecary and Man-Midwife, Upper Maudlin Lane.

WILLIAM HETLING, Surgeon, 18 Orchard Street.

NATHANIEL SMITH, Surgeon, 34 College Green.

JOHN BISHOP ESTLIN.

[1] The names are given in the order in which the signatures to the rules occur. The descriptions and addresses are from Mathews's *Bristol Directory* for 1807. In this the names of Mortimer and Lax are not given, but in the 1808 *Directory* Lax appears as "Surgeon & Apothecary, 11 Queen Square," and in that for 1809 there is an entry of "Berjew and Mortimer, Surgeons & Apothecaries, 17 Bridge Street." The name of Estlin, who was the son of the Rev. John Prior Estlin, Unitarian minister and master of a successful school at St. Michael's Hill, is not in the professional list of the 1807 *Directory*, but appears in that for 1809, when his residence is given as 2 Unity Street. Burroughs seems also to have had a branch establishment, for there is an entry in 1807 of "Yeo and Burroughs, *Apothecaries*, Granby House, Hotwells, and Portland Place, Clifton." Jermyn was one of the Surgeons at St. Peter's Hospital ; Spencer and Smith were two of the three "Extra Men-Midwifes" of the Dispensary.

On May 15th, 1807, Bowles, one of the surgeons at the Infirmary, died. On the following morning the *Bristol Mirror* contained the applications of ten surgeons for the vacancy, amongst whom were Jermyn, Daniel, Edgell, Lax, Hetling, and Smith. Apparently only three of the ten persisted in their candidature, and Hetling was elected with 395 votes, Lowe coming second with 167, and Smith third with 74. Another vacancy occurred shortly after, and Lowe was elected in July ; Daniel, Smith, and Edgell were among the unsuccessful candidates.

The members were to meet at one another's houses once a month [1] at half-past six o'clock. When the names were called over at seven, anyone then absent was to be fined one shilling. The fine for retaining a book longer than the time allowed was fixed at threepence each day. At the end of the year the books out of circulation were to be sold by auction, and any work not realising more than half its cost was to be taken at that price by the member who proposed it. It was considered necessary to insert in the rules that no druggist should be admitted into the Society, and it was laid down that no one should be elected a member except by a unanimous vote. One rule stated that "Each member shall keep an account of the books received by him and to whom forwarded, which account shall be regularly sent to the monthly meetings at or before seven o'clock." Omission to do this involved a penalty, apparently five shillings and half a crown at different times. The book in which the member was supposed to keep this record was afterwards known as "his green register," and notes about it frequently occur in the minutes and rules. [2]

The table which accompanies these notes gives the names of all the members [3] during the hundred years, together with the dates when they became, and when they ceased to be, members, and also shows the constitution of the Society at each change of membership. As the minute-book from April 21st, 1813, to January 20th, 1823, is missing, [4] there is some uncertainty about

[1] The day has varied from time to time.

[2] From the beginning of the Society a ledger was to be kept by the secretary for the entry of all books received, and by whom proposed and to whom and when they were sent. With the exception of a few years these entries are in existence.

[3] In a later number of the *Journal* it may be possible to give some biographical notes concerning these, and the Editor will be glad to receive anything of interest in connection with them. It will be fairly easy to obtain information about some of those who were fortunate enough to die before the issue of the *Dictionary of National Biography*, but about many it will be a matter of great difficulty to present anything like a connected account.

[4] It is impossible to say when the volume disappeared. It was not available in 1886, when the list of members was drawn up for the purpose of getting portraits of past members.

the dates of that period, and these are printed in *italic*. They may be taken as approximately correct, as the cash-book from 1817 and some of the fine-lists and sale-lists from 1818 to 1823 are in existence.[1] It will be noticed that only on rare occasions have the members been less than their full number for more than a short period. The Society has been so attractive, that men have often had to wait a long time for admission. There was no vacancy between 1894 and 1906.

The minute-books do not record much more than the election and absence of members and the names of the books proposed. It has been the custom for a long time for the secretary for the year to close his term of office by giving at the January meeting a dinner to the members. When this was introduced does not appear in the minutes.

It would naturally be expected that a society of enthusiasts such as those forming the Medical Reading Society would procure the most recent literature concerning any new development connected with the healing art. On January 20th, 1808, Willan's book on *Vaccine Inoculation*, published in 1806, was ordered.

It was not till February, 1808, that a twelfth member was proposed. Then Barton[2] was nominated, but at the following meeting his name was withdrawn, in consequence of a prior application made by Jermyn on behalf of Sheppard,[3] who, however, was not elected, because at the April meeting, after he had been twice balloted for, he did not receive a unanimous vote. At the June meeting, therefore, Barton was again brought forward, but met with the same fate as Sheppard after the vote had been twice taken. At the August meeting an acceptable member was found, when J. C. Swayne[4] was elected. From November till this date the Society had practically only ten members, as leave of absence had been granted to Estlin, who had gone to Edinburgh, and was away till this meeting.

Further interest in the vaccination question was apparent in

1 The cash-accounts are almost complete, but maay of the fine-lists and sale-lists are wanting.

2 "Charles Barton, Surgeon & Apothecary, 3 Hope Square."

3 "Godwyn and Sheppard, Surgeons, Redcliff Hill."

4 "John C. Swayne, Surgeon, &c., 15 Cumberland Street."

1809. At the June meeting Thomas Brown's *Inquiry into the Anti-variolous Power of Vaccination* (throwing doubts on its efficacy) was ordered, and in the following month the Society unanimously agreed to have (1) *The Report on Cow-pock Inoculation from the Practice of the Vaccine-Pock Institution*, by Pearson, Nihell, and Nelson, and any other statement by that Society; (2) *The Address of the Royal Jennerian Society*, instituted 1803; and (3) *A Statement of Evidence from Trials by Inoculation of Variolous and Vaccine Matter by the Physicians of the original Vaccine-Pock Institution, Established Dec.*, 1799,[1] printed in 1804. In January, 1810, the second edition of Bryce on the cow-pock was ordered.[2]

For facilitating the work of the member who had at the end of the year to compute the fines, an entry was always made of the absence of the "green register." At the meeting at Maurice's on May 26th, 1810, Estlin appealed against the fine being levied in his case, as "his book was on the table from seven to eight o'clock, and was then removed to another room in the house."

At this period, and for some time afterwards, it was the custom for the names of both present and absent members to be entered in the minute-book. For the benefit of the future historian of the Society, it is much to be wished that this practice should be restored, as it enables one to see at a glance the composition of the Society at any date. On December 21st, 1810, the cause of Jermyn's absence is stated to be "ill health," and on January 18th, 1811, both he and Spencer are among the absentees. There is no entry about the withdrawal of either of them, but as their names do not appear again, it may be taken for granted that this is about the date of their resignation. Crang[3] was elected in February and Baker[4] in March.

By rule of the Society, each original member paid a subscription of one guinea, and future members were in addition to pay an entrance fee of one guinea. Although there is no record

[1] The *Index-Catalogue*, vol. xv., 1894, p. 523, has 1779 by mistake.

[2] Some notes on the important books ordered by the Society during the hundred years would be of interest if space permitted.

[3] "Crang, James, Surgeon, &c., 17 Queen Square."

[4] "Baker, Robert, Apothecary, 3 St. James's Square."

of it in the minute-book, it would appear that at the beginning of 1811 the entrance fee was raised to two guineas, for in the accounts for that year the contributions of Crang and Baker are entered at three guineas each. At the annual meeting on January 17th, 1812, it was resolved "that the funds of the Society, after the payment of last year's accounts, should be equally divided amongst the respective members." This resulted in the payment of £4 5s. 11½d. to each member except Daniel, who, probably in correction of some error in his previous account, received £4 13s. 11½d. Daniel, who was the proposer of this distribution of the funds, resigned his membership in March, when Edgell also withdrew. At the next meeting Smith and Lax left the Society. It looks as if there was some rift in the lute, for then only five of the original members were left, and when, at the meeting in May, Crang withdrew, there had been five resignations in two months. At this May meeting a Committee, which had been appointed in the previous month, should have reported concerning the claims of the Society upon those gentlemen who had withdrawn, but there is no record in the minutes of their report, which no doubt was presented, as in the following December a special note was sent to Crang, who had refused to pay his fines.

The Society was evidently anxious at this time to have as members only those who would give it strength, for in May, when there were five vacancies, the name of William Maurice was withdrawn on account of his absence from the country. But Stock [1] and Prichard [2] were then elected, and at the June meeting Sheppard, who had failed at the ballot in April, 1808, was received into the Society; but although there were two vacancies, the Society would not have either Porter,[3] who had been proposed in May, or Perry,[4] who was balloted for in July; and the same fate awaited the younger Gold [5] on January 15th, 1813, on which

1 "Dr. J. E. Stock, 6 Park Street."

2 "Dr. J. C. Prichard, Berkeley Square."

3 Dr. W. Ogilvie Porter, 29 Portland Square, brother of Jane Porter who wrote *The Scottish Chiefs*.

4 "Perry, Chas. James, Surgeon and Apothecary, 13 North Street."

5 "Gold, Francis, Junr., Apothecary, 7 College Green."

date the resolution appears affirming the entrance-fee for new members at two guineas, although Prichard, Stock, and Sheppard were to be asked to pay only one guinea each.[1]

When the membership was only ten, the first minute-book closes with the meeting of April 21st, 1813. After this date reference should be made to the tabulated list for the dates of the election and departure of each member.

Whether any members were elected and withdrew during the ensuing four years it is impossible to say, for the next extant record of the Society is the statement of accounts for 1817, presented at the annual meeting on January 20th, 1818. In this are the names of William Swayne and Gold. The annual subscription was then half a guinea.

The sale-list of January, 1818, affords the information that the Society did not limit itself to medical literature, for it contains *The Quarterly Review, The Edinburgh Review*, and *The British Review.*

Between this date and March, 1823, when it was discontinued, the newspaper called *The Literary Gazette* had been ordered, and also the *Westminster Gazette*, for which a member held himself responsible.

When the rules were revised in 1820, the meetings were held on the third Saturday in every month from half-past six[2] till eight o'clock; the entrance fee was confirmed at two guineas, and the subscription was to be the amount necessary to defray the expenses.

Till 1823 it was a rule that the meetings should be held in the city; but on April 17th, when Hetling proposed to receive the Society either at his own house or at Reeves's Hotel,[3] it was resolved that the meeting should be at his house, 24 Royal York Crescent, to which he had just moved from 18 Orchard Street; and it was further resolved that as Goodeve was a resident in Clifton, living at 22 Mall, he should not be expected to receive the Society in Bristol. The distinction between Bristol and

[1] When Prichard rejoined the Society in 1832, he paid the entrance-fee of two guineas.

[2] Altered before 1823 to seven o'clock.

[3] Now the Turkish Baths.

Clifton seems to have been up to this time rigidly maintained, and it was not till the Reform Act came into force in 1832 that Clifton was added to the parliamentary borough. In 1835 it was included in the municipal area.

In December, 1823, the *Lancet*, the first number of which was dated Sunday, October 5th, 1823, was ordered from the commencement, but in the following February "it was resolved that the *Lancet* is a publication unfit for this Society, and that it be discontinued."

The fine for non-attendance, after having been increased at some date not discoverable to two shillings for absence during the whole meeting, was reduced to one shilling in January, 1825; and in the following June the Society re-considered its action in reference to the *Lancet*, and ordered it in half-bound volumes, giving the impression that thus the members would receive less contamination than by touching the unclean thing in weekly numbers. In September Howell,[1] Wilson,[2] and Nathaniel Smith[3] "were balloted for as members, and not received."

The two shilling fine for non-attendance at eight o'clock was restored in January, 1826, when the Society determined to take the *Lancet* again in numbers, and also to be responsible for the *Westminster Review*.

The dissatisfaction of the Society with the *Lancet* was again in evidence in August, 1828, when a proposition was carried that "This Society, considering the *Lancet* as a publication injurious to the respectability and best interests of the profession and disgraceful to the medical men who conduct it, resolves that its circulation in the Society be henceforth discontinued."

In 1829 it was decided to give up the *Quarterly*, *Edinburgh*, and *Westminster Reviews*.

At the annual meeting in January, 1831, the hour of meeting was altered to eight instead of seven, and in 1834 the day was

1 Dr. John Howell, living at 45 Royal York Crescent, was one of the physicians at the Clifton Dispensary, then at 1 Dowry Square.

2 Wilson's name first appears in the *Directory* for 1826 in partnership with Mortimer at 17 Bridge Street.

3 He had retired in 1812, and was evidently seeking re-election.

changed to the first Wednesday of each month, and it has remained so till the present time.

At this time it was resolved to take again the *Literary Gazette*, which the Society had been without since March, 1823.

An attempt to reintroduce the *Lancet* failed in January, 1835, but was successful at the next annual meeting in 1836, when it was also decided to subscribe for the *British and Foreign Quarterly Review*.

A rule that no accumulated fines on a book should exceed one half of its prime cost was carried in January, 1837, and in the following year it was decided to abolish the second fine of one shilling imposed on absentees from the meetings, but at the next annual meeting it was again restored.

It was unanimously resolved on January 8th, 1840, to discontinue the "Green Register."[1] At the next annual meeting the Society ordered the *Provincial Medical and Surgical Journal*,[2] but resolved again to give up the *Literary Gazette*.

Nothing of importance is recorded in the minutes from this time till January, 1846, when the following propositions were carried unanimously: (1) That Sunday be a "*dies non;*" (2) That the days of transfer be Monday and Thursday, and that the period of detaining a book be always three days or a multiple of three days; (3) That the Green Register be restored.[3] It was also determined once more to give up the *Lancet*.

In January, 1847, it was resolved "that Mr. Coates [who had resigned in 1837] be allowed to read the books when out of circulation upon the payment of one guinea per annum."

In January, 1848, the *Lancet* seems to have been again taken on the condition of one member being responsible for it. At the April meeting we can imagine that a lively discussion took place,

[1] See p. 224.

[2] The precursor of the *British Medical Journal*.

[3] Among the Society's books are Morgan's register from 1846 to the time he left the Society, in 1872; George Hetling's from January, 1846, to December, 1847, and in the same volume William Cross's from January, 1848, to January, 1870; Estlin's from May, 1846, to December, 1854, together with that of Hore, who succeeded him, from May, 1855, to December, 1870; Brittan's from May, 1865, to November, 1873.

for it is recorded that three gentlemen, "tho' 3 minutes after time by the institution clock, pleaded being in time by their watches, and it was determined by 5 to 4 that they should not be fined."

In the minutes of February, 1849, there is a vague record concerning "Nathaniel Smith, who was proposed and seconded," but there is no note of his rejection, and he was certainly not elected. As he had failed at the ballot in 1825, this was his second unsuccessful attempt to re-enter the Society, from which he had withdrawn in 1812. The vacancy for which he was nominated was not filled till March, 1850.

In 1854 the Society again took the responsibility of the *Lancet*, as the member who had proposed it in 1848 declined any longer to have it at half-price, but as a member was willing upon that condition to take the *Quarterly Review* and the *Edinburgh Review*, they were again circulated in the Society.

The *Lancet*, however, remained in favour with the Society but a short time, for in April, 1854, it was resolved "That in the opinion of the members of this Society the conduct of the *Lancet* of late (more especially with reference to the proceedings in the case of Mr. Gay at the Royal Free Hospital) has not been such as becomes the Journal claiming to be the organ of an enlightened and honorable profession—and therefore, that it be discontinued." The circumstances were connected with the dismissal of the well-known surgeon, John Gay, from the Hospital in December, 1853. This caused much indignation among many members of the profession, and a meeting[1]—at which it was suggested that Thomas Henry Wakley, son of the editor of the *Lancet*, and who was one of the surgeons at the Hospital, had had something to do with it—was held on January 18th, 1854, to protest against the action of the committee of the Hospital. The pages of the *Lancet* for the first half of 1854 are amply provided with very strong language on this subject.

[1] The secretary of the organisation of this meeting was Harvey Ludlow, brother of Ebenezer Ludlow, successively Resident Medical Officer and first Assistant-Physician at the Bristol Royal Infirmary. The Royal Medical and Chirurgical Society, at its meeting on March 1st, 1854, also decided to withdraw the *Lancet* from its list.

On January 10th, 1855, there is a note that "Mr. Swete[1] was unanimously elected an honorary member as successor to Mr. Coates."[2] No reason is stated for the choice of Mr. Swete, who had never been in the Society. There is nothing in the revised rules of 1820 or in the 1877 edition about honorary members; but a resolution may have been passed between 1820 and 1823 in reference to them, and this may have been in evidence at the time, although the minute-book for that period has been lost. Coates had resigned in 1837 after a membership of less than five years, and there is no record why the special privilege had been conferred upon him. At this January, 1855, meeting Estlin, who had been in the Society more than forty-seven years, resigned, and he was very properly made an honorary member.[3] Upon this occasion the sins of the *Lancet* had been partly condoned, and only eight months after the emphatic resolution condemning it, it was again ordered for the Society, but only on the undertaking of a member to purchase it at the sale at half-price.

At the October meeting in 1855, the Society, having to choose between William Budd and Sawer, elected the former.

The annual meeting in January, 1856, decided that the Society should take the *Lancet.*

A proposal that the Society should no longer circulate the *Quarterly Review* and the *Edinburgh Review* failed in January, 1857, to find a seconder; and at the next annual meeting the *Athenæum* was added to the list on the same terms as the *Reviews* were taken in 1854, but it remained only for twelve months. The same member who had proposed the discontinuance of the *Reviews* failed again in 1857 to secure it. In March it was resolved

[[1] "E. H. Swete, surgeon, 1 Dowry Parade," the author of *Flora Bristoliensis*, 1854, on the title-page of which he is described as "Lecturer on Botany at the Bristol Medical School." A search through the minute-books of the School has failed to discover any entry made about him.]

[2] The title of honorary member was a misnomer, as Coates was required to pay an annual subscription (see p. 230). The resolution of January, 1847, gave him no distinctive title. Mr. Swete paid a guinea a year for two years.

[3] Smerdon, who had been "acting secretary" for thirty years, was made an honorary member upon his resignation on account of illness in 1870. Crosby Leonard was elected an honorary member in July, 1879, after a membership of nearly twenty years, but he lived to enjoy the honour only a few months.

" that Mr. Goodeve be an associate of the Society," on the understanding that he " should be liable for an annual subscription, but not for fines ; " but the minutes afford no information as to the reason of this step, which was no doubt taken on account of his long membership.[1] Probably the connection by associateship, for which there seems no provision in the rules, was a merely nominal one, although, as he was not to be fined, it would appear that he was to receive the books, perhaps after they had gone the round of the members.

The Society declined in January, 1860, to add *Bentley's Quarterly Review*[2] to the list, and in 1861 it decided to give up the *Quarterly Review* and *Edinburgh Review*, which, however, were restored in 1864.

A new departure is chronicled in July, 1864, when a subcommittee was appointed " to make arrangements for the excursion," and in June, 1865, two members were requested " to arrange for the annual expedition." A member of the Society at the time recollects going to Aust, where they dined and geologised, but no information is forthcoming in reference to the other outing, and probably there were only these two.

The desire for high-class periodical literature other than medical was frequently manifested, and in January, 1869, the *Revue des Deux Mondes* was ordered, but remained on the list only till January, 1870, when with the *Quarterly Review* and the *Edinburgh Review* it was discontinued.

Social changes made it desirable to alter the hour for meeting, and in January, 1871, it was decided to make this nine o'clock instead of eight, and at that hour it has since remained. And on December 4th, 1872, it was agreed that the annual meeting should be held in December instead of January, thus giving more opportunity for the transaction of business than on the evening of the dinner. In the revised rules, which were issued in 1877, the January meeting is called the annual meeting, but in November, 1878, the resolution of December 4th, 1872, was re-adopted.

In January, 1881, the rule referring to the fine-committee

[1] Goodeve had resigned in the previous August after having been a member for 38 years.

[2] This died after its second volume.

was elaborated with much detail, and in 1882 some minor alterations about the election of secretary were adopted.

In order to facilitate the ordering of books, it was agreed in 1883 to take in the *Bookseller*, a monthly trade-journal giving classified lists of new publications, and it was resolved that the secretary should produce it at each meeting; but this useful periodical seems to have been in favour for only one year.

In 1884 the Society decided to make an effort to procure the portraits of all past members. This has succeeded to some extent, and they are preserved in albums among the archives of the Society.[1]

Greig Smith, at his secretarial dinner in 1885, embellished the *menu* card with some lines of verse.[2]

In 1891 the Library of the Bristol Medico-Chirurgical Society was opened in the Literary and Philosophic Club, and was moved to the Medical Department of University College in 1893. The Reading Society, at its meeting in January, 1902, nobly gave all its periodicals to the library, but in 1904 they "were presented to those members wishing to have them."

At the meeting on March 7th, 1907, "it was unanimously

[1] The following have not yet been obtained, and the Society would be grateful for any help in securing them.

| | | | |
|---|---|---|---|
| Jermyn | Gold | Stock | Mortimer |
| B. Spencer | J. Maurice | Bernard | Goodeve |
| Daniel | Gilby | W. Maurice | Hore |
| Edgell | Sheppard | Howell | Ring |
| Lax | Baker | King | |

Arrangements would be made for photographing any portraits that may be lent for the purpose.

---

[2] ESTO MIHI, ERO TIBI.

Twelve Medicos of high renown,

In this our ancient Western Town,

Harmoniously combine

To take in books for culture's sake,

Meet once a month for tea and cake,

And once a year to dine.

These twelve, of varied reputation,

Are competent to treat a nation

For, be your ailment what you please,

There 's one at least for your disease.

Of eyesight should you threaten loss,

The man to make you see is **Cross**;

And should your reason show a flaw,

The man to lock you up is **Shaw**;

And if you think you cannot hear,

Let **Harsant** peep into your ear.

From hidden holes your germs to ferret

There 's none so 'cute as **Markham Skerritt**:

His microscope will soon determine

How **Shingleton** will kill the vermin;

And livers weary of their life

Find comfort in the arms of **Fyffe.**

With **Griffiths** strong on vaccination

And apt Shakespearian quotation,

With **Lansdown** for our angiomas,

And **Dobson** for round-celled sarcomas,

We need not fear: but if more ill,

There 's **Beddoe** and there 's **Prichard** still.

Should these eleven fail to mend you,

Then **Greig Smith's** knife will gently end you.

There is no information about the Secretaries for 1814, 1815, 1816, 1821, and 1822. I have assumed that the older members who had not been secretaries were so in turn, although the handwriting of the cash accounts for 1820–22 is evidently the same as that for 1819.

| Period of Membership. | | Members and their Secretarial Years. | |
|---|---|---|---|
| 1807—1811 | | Mr. THOMAS JERMYN | (1807) |
| 1807—1812 | 1 | Mr. HENRY DANIEL | (1809) |
| 1807—1812 | | Mr. RICHARD EDGELL | (1811) |
| 1807—1811 | | Mr. BENJAMIN SPENCER | |
| 1807—1849 | | Mr. WILLIAM MORTIMER | (1818, 1835) |
| 1807—1812 | | Mr. ROBERT LAX | (1808) |
| 1807—1822 | | Mr. BENJAMIN GUSTAVUS BURROUGHS | (*1814*) |
| 1807—1819 | | Mr. JOSEPH MAURICE | (*1815*) |
| 1807—1837 | 2 | Mr. WILLIAM HETLING | (1812, 1817, 1832) |
| 1807—1812 | 3 | Mr. NATHANIEL SMITH | (1810) |
| 1807—1855 | 4 | Mr. JOHN BISHOP ESTLIN | (*1816*, 1838) |
| 1808—1847 | 5 | Mr. JOHN CHAMPENY SWAYNE | (1820, 1837) |
| 1811—1812 | | Mr. JAMES CRANG | |
| 1811—1825 | | Mr. ROBERT BAKER | (1813) |
| 1812—1839 | 6 | Dr. JAMES COWLES PRICHARD | (1819, 1833) |
| 1812—1830 | 7 | Dr. JOHN EDMONDS STOCK | *1821*) |
| 1812—1824 | | Mr. EDWARD W. SHEPPARD | (*1822*) |
| *1814*—1819 | 8 | Mr. FRANCIS GOLD | |
| *1814*—1825 | 9 | Mr. WILLIAM SWAYNE | (1823) |
| 1819—1820 | | Dr. WILLIAM GILBY | |
| 1820—1835 | | Mr. WILLIAM MAURICE | (1824) |
| 1820—1858 | 10 | Mr. WILLIAM JAMES GOODEVE | (1825, 1842, 1850) |
| 1823—1859 | | Mr. ISAAC LEONARD | (1826, 1843, 1855) |
| 1824—1843 | | Mr. JOHN KING | (1827) |
| 1825—1872 | 11 | Mr. WILLIAM FREDERICK MORGAN | (1828, 1847, 1863) |
| 1825—1832 | | Dr. CHARLES EDWARD BERNARD | (1829) |
| 1828—1832 | | Mr. JOHN GRANT WILSON | (1830) |
| 1830—1845 | | Mr. JOHN HARRISON | (1831) |
| 1832—1837 | | Mr. WILLIAM COATES | (1834) |
| 1835—1870 | | Mr. CHARLES SMERDON | (1836, 1853, 1867) |
| 1837—1837 | | Dr. JOHN HOWELL | |
| 1838—1848 | 12 | Mr. GEORGE HILHOUSE HETLING | (1839) |
| 1838—1844 | 13 | Mr. WILLIAM BENJAMIN CARPENTER | (1840) |
| 1839—1875 | | Dr. ALEXANDER FAIRBROTHER | (1841, 1854, 1868) |
| 1844—1856 | 14 | Mr. JOHN COLTHURST | (1844) |
| 1844—1885 | 15 | Mr. AUGUSTIN PRICHARD | (1845, 1864) |
| 1845—1858 | 16 | Mr. JOSEPH GRIFFITHS SWAYNE | (1846) |
| 1847—1858 | 17 | Mr. ROBERT WILLIAM COE | (1848, 1852) |
| 1848—1875 | | Mr. WILLIAM CROSS | (1849, 1865) |
| 1850—1855 | | Mr. EDWARD WALDO | (1851) |
| 1855—1871 | | Mr. HENRY AUGUSTUS HORE | (1856, 1869) |
| 1855—1869 | 18 | Dr. WILLIAM BUDD | (1857) |
| 1856—1877 | 19 | Mr. THOMAS GREEN | (1858, 1873) |
| 1858—1865 | | Dr. HENRY EDWARD FRIPP | (1859) |
| 1858—1873 | 20 | Dr. EDWARD LONG FOX | (1860) |
| 1858—1885 | | Dr. JOHN BEDDOE | (1861) |
| 1859—1879 | 21 | Mr. CROSBY LEONARD | (1862) |
| 1865—1882 | 22 | Dr. FREDERICK BRITTAN | (1866) |
| 1869—1878 | 23 | Mr. ROBERT WILLIAM TIBBITS | (1870) |
| 1870—1876 | | Mr. EDMUND COMER BOARD | (1871) |
| 1871—1873 | 24 | Mr. THOMAS EDWARD CLARK | (1872) |
| 1873—1877 | | Dr. WILLIAM HENRY SPENCER | (1874) |
| 1874— | | Dr. ROBERT SHINGLETON SMITH | (1875, 1890) |
| 1874—1892 | | Mr. NELSON CONGREVE DOBSON | (1876, 1891) |
| 1876—1893 | | Mr. FRANCIS POOLE LANSDOWN | (1877) |
| 1876—1892 | | Mr. LEMUEL MATTHEWS GRIFFITHS | (1878) |
| 1876— | 25 | Dr. EDWARD MARKHAM SKERRITT | (1879, 1898) |
| 1877—1881 | | Mr. CHARLES GORE RING | (1880) |
| 1877— | | Dr. JOHN EDWARD SHAW | (1881, 1899) |
| 1879—1885 | 26 | Dr. WILLIAM JOHNSTONE FYFFE | (1882) |
| 1879—1892 | | Mr. FRANCIS RICHARDSON CROSS | (1883) |
| 1881—1886 | 27 | Mr. JAMES GREIG SMITH | (1884) |
| 1882—1906 | | Mr. WILLIAM HENRY HARSANT | (1885, 1900) |
| 1885—1893 | | Dr. ARTHUR BANCKS PROWSE | (1886) |
| 1885—1891 | | Mr. CHARLES FREDERICK PICKERING | (1887) |
| 1885—1906 | | Mr. ARTHUR WILLIAM PRICHARD | (1888, 1901) |
| 1886— | | Dr. JOHN MICHELL CLARKE | (1889, 1902) |
| 1891— | | Mr. JAMES PAUL BUSH | (1892, 1903) |
| 1892— | | Mr. JOHN DACRE | (1893, 1904) |
| 1892—1893 | | Dr. PATRICK WATSON WILLIAMS | |
| 1892— | | Dr. GEORGE PARKER | (1894, 1905) |
| 1893— | | Dr. JAMES SWAIN | (1895, 1906) |
| 1894— | | Dr. ROBERT GUTHRIE POOLE LANSDOWN | (1896) |
| 1894— | | Dr. BERTRAM MITFORD HERON ROGERS | (1897) |
| 1906— | | Mr. JAMES TAYLOR | (1907) |
| 1906— | | Mr. GEORGE MUNRO SMITH | |

1 See *Bristol M.-Chir. J.*, 1890, viii. 186–7.

2 See *Bristol M.-Chir. J.*, 1890, viii. 182–3; 1892, x. 268.
3 See *Bristol M.-Chir. J.*, 1890, viii. 187–8.
4 See *Dict. Nat. Biog.* and Augustin Prichard's *A Few Medical and Surgical Reminiscences*, 1896, pp. 10—14.
5 See *Bristol M.-Chir. J.*, 1892, x. 269—70, 289—90.
6 Resigned in 1825, but was re-elected in 1832. See *Dict. Nat. Biog.*; *Bristol M.-Chir. J.*, 1890, viii. 176–8; 1892, x. 265; Augustin Prichard's *A Few Medical and Surgical Reminiscences*, 1896, pp. 14—16, 25
7 See *Bristol M.-Chir. J.*, 1890, viii. 172–4.
8 See Latimer's *Annals of Bristol in the Eighteenth Century*, 1893, p. 524.
9 See *Bristol M.-Chir. J.*, 1890, viii. 169—70.

10 See Latimer's *Annals of Bristol in the Eighteenth Century*, 1893, p. 524.

11 See *Bristol M.-Chir. J.*, 1890, viii. 170.

12 See *Bristol M.-Chir. J.*, 1890, viii. 183–4; 1892, x. 272.
13 Became M.D. in 1839. See *Dict. Nat. Biog.* and Augustin Prichard's *A Few Medical and Surgical Reminiscences*, 1896, pp. 30–2.
14 See *Bristol M.-Chir. J.*, 1892, x. 272.
15 See his *Few Medical and Surgical Reminiscences*, 1896, and *Some Incidents in General Practice*, 1898; *Bristol M.-Chir. J.*, 1892, x. 272; 1898, xvi. 1—15.
16 Became M.D. soon after joining. See *Bristol M.-Chir. J.*, 1892, x. 272; 1903, xxi. 193—202.
17 Resigned in 1848, upon leaving England, but was re-admitted in 1850. The end of the secretarial work for 1848 was taken by Hetling. See *Bristol M.-Chir. J.*, 1892, x. 273.

18 See *Dict. Nat. Biog.*; *Bristol Royal Inf. Reports*, 1878—79, i. 361–7; *Bristol M.-Chir. J.*, 1892, x. 273.
19 See *Bristol M.-Chir. J.*, 1892, x. 272.
20 See *Bristol M.-Chir. J.*, 1892, x. 273; 1902, xx. 97—105.

21 See *Bristol Royal Inf. Reports*, 1878—79, i. 210—25, 342—60; *Bristol M.-Chir. J.*, 1892, x. 273.
22 See *Bristol M.-Chir. J.*, 1891, ix. 67—70; 1892, x. 273.
23 See *Bristol Royal Inf. Reports*, 1878—79, i. 226—31.

24 Became M.D. in 1872. See *Bristol M.-Chir. J.*, 1892, x. 287.

25 Died April 29th, 1907. See *Bristol M.-Chir. J.*, 1907, xxv. 97—108.

26 See *Bristol M.-Chir. J.*, 1901, xix. 97—100.

27 See *Bristol M.-Chir. J.*, 1897, xv. 105—21.

carried that to celebrate the centenary of the Society, a dinner should be held on the day of meeting nearest to the date of inauguration of the Society," and that past members be invited as guests of the Society. In accordance with this resolution the twelve members of the Society and six former members dined together at the Clifton Club on April 3rd.[1]

Although the limited information afforded by the records has made it impossible to provide anything like an adequate history of the Society, it would be wrong to close this fragmentary account without giving a full meed of praise to it for the indirect benefit which it has conferred upon the local profession, whose indebtedness to it should be distinctly recognised. During the long period of a hundred years a small Society, numbering many scholarly and prominent men, has shown the necessity, in keeping abreast of the times, of having constantly before it the best literature obtainable; and its continuance is evidence that the needs of an enlightened profession are not entirely met by the provision of an excellent reference library, such as local medical practitioners have at their command, but that it is essential that there should be the means of consulting desirable books and periodicals at more leisure than is possible with a library which is not a lending one.

The Society, now so strongly representative of all that is best and highest in the profession, and with a century's good work as its voucher, could, by taking the initiative in the founding of a medical institute or club that would bring together practitioners in closer professional and social relationship, add to the usefulness which hitherto it has been able to achieve. Such an institution should elicit the practical sympathy and hearty co-operation of the local profession, members of which should see in it an opportunity for mutual help and encouragement in their difficulties and disappointments; and the Society would have the privilege of extending, in an ever-widening circle, the purpose of "promoting medical knowledge and friendly intercourse," which its originators set before themselves as their object, and which succeeding generations have so well and so honourably maintained.

[1] A photograph of the occasion is preserved in the minute-book.

